# Effects of Defense Suppliers’ Practice of Online Character Education on the Employees’ Learning Motivation and Perception of Integrity During COVID-19

**DOI:** 10.3389/fpsyg.2021.771124

**Published:** 2021-10-22

**Authors:** Hong-Chin Hsiao

**Affiliations:** Department of Public Policy and Management, I-Shou University, Kaohsiung, Taiwan

**Keywords:** defense supplier, online character education, learning motivation, perception of integrity, affective components

## Abstract

The 21st century marks a period where the pursuit of innovation, the advance of information media, and diverse values have gained great significance. In the face of information, economic impacts, and other challenges, developed countries in particular have come to emphasize online character education, as cultivating the people’s character to have a positive attitude toward life. Teaching people desirable core values and moral thinking to cultivate pleasant character could enhance personal happiness and social harmony, boost national competitiveness, and be the key to a more harmonious society in the future. Education is the stable rock of ethics and morality, and like the foundation upon which to build a house, an unstable rock would result in the danger of collapse. People’s sense of propriety, justice, integrity, and honor, as well as morality, is in decline. In such a social climate, online character education plays an extremely important role. Taking employees of defense suppliers for the Ministry of National Defense, Taiwan, as empirical subjects, 226 employees proceeded with our 20-week (4 h per week, for a total of 80 h) experimental teaching research. The research results reveal that: (1) online character education would affect learning motivation, (2) online character education would affect the perception of integrity, and (3) learning motivation presents significantly positive effects on the perception of integrity. Our results suggest that online character education and discussion is expected to help defense suppliers cultivate good social interaction skills and character to build harmonious interpersonal relationships.

## Introduction

Rapid change in society, changes in family structure such as the increase in single-parent families, and the decline of traditional family functions can cause non-standard behavior and challenging personalities as entire social norms and value structures are being relaxed or weakening. In view of the modern change in social values, social problems increase much more than in past years. Furthermore, adolescent delinquent behaviors are fast becoming a headache for parents and teachers, as school education is left to deal with this great crisis. The spirit of respect for teachers disappears, with many teachers complaining regarding the difficulty in being teachers, and discipline wayward adolescent, but their hands are tied. In terms of “character and education,” the loss of positive character is most alarming and worrying. Education is the bedrock of ethics and morality, and much like the foundations of a house, a solid structure reduces the danger of collapse. People’s sense of propriety, justice, integrity, and honor, as well as morality are in decline nowadays. Online character education plays an extremely important role in such a social climate. Hence, the 21st century is a time where the pursuit of innovation, the advance of information media, and diverse values gain paramount importance. In the face of information and economic impact and challenges, developed countries in particular stress on online character education, as cultivating the people’s sound character to have a good attitude toward life and teaching the people positive core values and moral thinking could cultivate good character and create personal happiness and social harmony, boost national competitiveness, and be the key in a more harmonious society in the future. The advance of information media results in sex, and violence, and utilitarian values on the media negatively affect people’s character. Current education presents excessive formalization and doctrine but ignores the value of moral education. It is therefore important to plan online character education curricula, teach people positive moral thinking, and cultivate a sound personality to promote people’s character and encourage a benign climate within society.

In recent years, online learning has been flourishing. It has been almost 2 years since the major outbreak of COVID-19. Technological advancements have allowed online teaching to become the new normal. Distance teaching includes online teaching, e-learning, and online learning. It means that teachers and students interact with each other through the communication network, computer network, and video calls. Students are not confined to time and place since they don’t have face-to-face lectures. The courses could be asynchronous or synchronous and therefore secure undeniable advantages concerning immediacy, convenience, and variety. Many people’s lives are affected by the pandemic. Some saw their vacations, business trips, or weddings canceled, while others became exposed to the risk of job loss, suffering pay cuts, or being forced to take unpaid leave. In this period, personal safety protection is the immediate priority. Nauzeer and Jaunky (2021) segmented online moral education into four levels; The third level “sincere” comes from self-discipline, enterprising, introspection, gratitude, and perseverance. These qualities can improve the ability for self-improvement and respect, understanding, and appreciation of others (Bialik et al., 2015). On the other hand, Brooks (2001) believed that ten character traits should be prioritized, including public morality, cooperation, responsibility, help, respect, care, justice, trust, gratitude, and introspection. As stated above, the research of the core values around introspection and self-reflection is deficient (Djiwandono, 2016). For this reason, the effects of defense suppliers’ practice of online character education on their employees’ learning motivation and perception of integrity are discussed in this study, and are expected to help defense suppliers cultivate good social interaction skills and character to build harmonious interpersonal relationships.

## Literature Review and Hypothesis

Broadly speaking, Hua (2019) regarded online character education as all activities preceded in informal curricula, aiming to teach students to become good-hearted people. Narrowly, online character education referred to special character training, teaching by matching special values and learning activity with children’s nature and learning styles, to effectively enhance students’ learning motivation. Rissanen et al. (2018) proposed that online character education should develop the relationships of respect, openness, and caring for teachers and students, provide various opportunities and design situations with plans for implementing morality, and positively encourage students’ performance to enhance learning motivation and effectively facilitate morality growth in order to establish harmony within society and the basis of advancement. Phillippi and Lauderdale (2018) described online character education as the process to educate students to enhance individual good traits, recognize virtue, learn virtue, and do well, as well as internalize good habits. Therefore, recognizing virtue, appreciating virtue, doing good, and cultivating positive morality through education and learning allowed students to present behaviors conforming to social norms and value identity, as well as to demonstrate self-reflection skills to effectively enhance their learning motivation. The following hypothesis is therefore proposed in this study:

H1: Online character education would affect learning motivation.

Pintrich (1989) stated that the practice of character education through schooling, family, and social education allows students to know good and do good. They will be able to internalize honesty and turn it into a habit. It is also the process of cultivating students to conform to social morality standards. It stresses the process of having students perceive the value of character from experience and further practice character, expecting students to be able to spontaneously practice good character in daily life as the final goal. Drake and Reid (2018) mentioned that online character education, through the teaching and learning process, facilitated individuals to develop ethical responsibility and caring, and by teaching students important core ethical values, effected the promotion of interpersonal relationships with caring, honesty, responsibility, and mutual respect. Sari (2019) indicated character education is defined as a direct oral method to inform what can be done and what cannot be done and to teach children to obey and practice. Besides, children’s behaviors are directly supervised with lessons. It is important to teach children to get used to compliance, honesty, benevolence, sincerity, and trust for their positive development. Accordingly, the following hypothesis is therefore proposed in this study.

H2: Online character education would affect the perception of integrity.

Liao and Hsu (2019) discovered that the positive effects of intrinsic goal orientation, work value, self-efficacy, expected success, and locus of control in learning motivation on academic performance showed notable correlations with the perception of integrity. Pohan and Malik (2018) indicated that learning motivation could facilitate pupils to continuously and positively pursue online character education performance, interpersonal relationships, and assistance from others in acquiring successful experiences for self-affirmation and the establishment of the perception of integrity. Soleimani and Lovat (2019) mentioned that students would show different learning motivations according to past performance experience, vicarious experiences, verbal persuasion from others, and emotional responses; from the observation of peers’ learning, online character education could guide students to establish self-confidence through successful learning experiences and provide students with support and affirmation in the learning process, as well as positively create good learning situations to have students know and understand the process and affect the learning motivation to enhance the perception of integrity (Wall and Leckie, 2017). In this case, the following hypothesis is proposed in this study.

H3: Learning motivation shows significant and positive effects on the perception of integrity.

## Methodology

**FIGURE 1:**
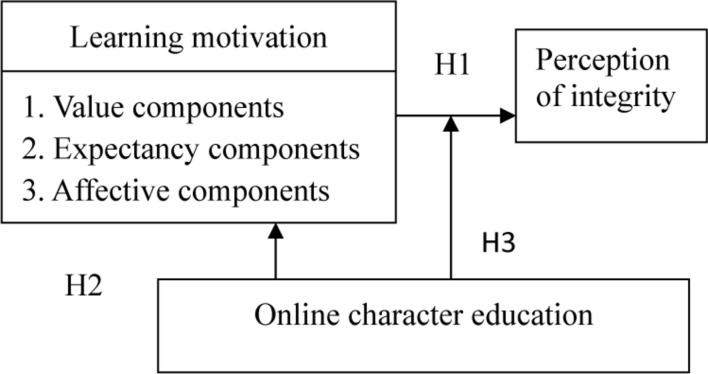
Conceptual framework.

From the above literatures, it is considered in this study that there is relevance among online character education, learning motivation, and integrity perception. The influence path and the hypothesis test are therefore organized in this study as Figure 1.

### Measurement of Research Variable

#### Learning Motivation

Referring to He (2019), students’ motivation in the learning process constitutes the components of value, expectancy, and affection.

1. Value components: Referring to students’ belief in the importance and value of engaging in the learning activity, including goal orientation and work value. The components of value include intrinsic goal orientation, extrinsic goal orientation, and work value.
2. Expectancy components: Referring to students’ belief in the ability to complete learning and smoothly achieve the expectancy of learning, including learning self-efficacy, expected success, and locus of control.
3. Affective components: Referring to the feeling and emotional response to personal ability in the learning process and result.

#### Perception of Integrity

The Honesty Scale proposed by Feng (2019) is used for measuring the perception of integrity in this study.

### Research Subject and Sampling Data

Taking employees of defense suppliers for the Ministry of National Defense, Taiwan, as the empirical subjects, 226 employees proceeded with the 20-week (4 h per week, for a total of 80 h) experimental teaching research. The data are analyzed with SPSS, and factor analysis, reliability analysis, regression analysis, and analysis of variance are utilized for testing various hypotheses.

### Analysis Method

The goodness-of-fit test with the LISREL model is generally measured from overall model fit (i.e., external quality of model) and the internal quality of the model. The common fit indices for the test of overall model fit contain (1) “χ^2^ ratio” (Chi-Square ratio), standing for the difference between the real and theoretical model and the expected value, which is better if smaller than three, (2) the closeness of the goodness of fit index (GFI) and adjusted goodness of fit index (AGFI) to one shows the better fit, (3) root mean square residual (RMR), reflecting the square root of “fit of residual variance/covariance mean,” which is better when smaller than 0.05, and (4) the incremental fit index (IFI) if higher than 0.9 reveals good model fit.

### Reliability and Validity Test

Validity refers to the measurement scale being able to measure what a researcher intends to measure. General types of validity include “content validity,” tending to qualitative verification, “criterion validity,” using known external criterion and the correlation coefficient of the test for the evaluation, and “construct validity,” for evaluating the measurement of the theoretical consistency of the measurement to other observable variables. The questionnaire content in this study is based on past theories and refers to the real situations of research subjects to design a measurement tool to truly express the essence of affairs with complete representativeness to ensure the validity of the content. Moreover, the final commonality estimate of factor analysis results is applied to test the construct validity of items, and the obtained validity appears in 0.8∼0.9, revealing a good validity test of the questionnaire in this study.

## Empirical Results

### Model Fit Test

“Maximum Likelihood” is utilized in this study for the estimation, and the analysis results achieve convergence. Results illustrate that the model fit indices in Table 1 pass the test, fully reflecting the good external quality of the model.

**TABLE 1:** Model analysis result.

Overall fit	Evaluation indicator	Judgment standard	Result
	*p*-value	*p*-value > 0.05	0.000
	χ^2^/d.f.	<3	1.755
	GFI	>0.9	0.963
	AGFI	>0.9	0.911
	CFI	>0.9	0.946
	RMR	<0.05, <0.025 excellent	0.017
	RMSEA	0.05∼0.08 good	0.042
		<0.05 excellent	
	NFI	>0.9	0.926
	IFI	>0.9	0.915

### Test of Path Relationship

In terms of the test of the quality of the internal model, the squared multiple correlation (SMC) of manifest variables is higher than 0.5, revealing good measurement indicators of latent variables. Furthermore, latent variables of online character education, learning motivation, and integrity perception show a component reliability higher than 0.6, and the average variance extracted of dimensions is higher than 0.5, apparently conforming to the requirement for the internal quality of the model.

The model analysis results reveal positive and significant correlations between online character education and learning motivation (0.846), learning motivation and integrity perception (0.871), as well as online character education and integrity perception (0.863) that H1, H2, and H3 are supported.

## Discussion

The research results reveal the importance of online character education. Defense administrative units could invite online character education professional communities and teams of teachers in various fields to discuss the promotion of online character education in defense units, design online character education-oriented curricula, and select material suitable for defense units to facilitate a conducive atmosphere for online character education to enhance the effectiveness of online character education. Character education teachers could utilize social resources, such as online character education resource platforms, teaching plans in the papers in the National Digital Library of Theses and Dissertations in Taiwan, online character education materials in Rainbow Family, Dandelion magazine, Jing Si Aphorisms, Observing Merits, and Appreciating Kindness, films on YouTube, advertisements, or news, to reduce lesson preparation time and the efforts of designing lesson plans and to achieve good learning effectiveness using half the effort for twice the result. Tyra (2012) explained that national defense suppliers should promote their activities and campaigns during leisure or meeting time according to the core value of the current month. They should promote online moral education and develop a good moral environment to deepen the impressions of employees working in national defense administrative departments. This would be helpful for those employees in real life as well (Ong’ong’a, 2021). According to the core value of the current month, they will be able to find articles about relative events and people. Reading examples could be a way to deepen employees’ cognition and approval about the current online moral education. By internalizing the knowledge, it can enhance the moral literacy of employees in national defense suppliers and create a working environment with a superior moral culture (Peterson, 2020). Well-designed databases could provide the latest and most accurate information access routes to solve the problems in obtaining teaching materials in cases where specific practice or training appears to be lacking. Connecting with relevant teaching resources and online character education promotion and successfully integrating these relevant resources and materials could provide reference of normative samples for successive defense units or teachers.

## Conclusion

Due to the pandemic outbreak, many countries in the world had to lock down their cities and restrict their citizens’ daily activities under strict conditions in 2020. Since distance learning at home is a temporary solution, online learning became a trending topic overnight. This research concentrated on how national defense suppliers implementing online character education could have impacts on their employee’s learning motivation and perception of integrity. The research findings show that employees of defense suppliers learn to be honest, cultivate good and healthy habits, commit to promises, correct mistakes, be self-confident, do things with heart, are in control of their emotions, treat others with politeness, are helpful and cooperative, know to share, and show consideration to family members as well. From the feedback, all employees of defense suppliers give positive affirmation, presented positive changes after participating in the online character education activity, and appreciate the value of online character education. In the online character education process, teamwork is applied to establish the peer relationship among employees of defense suppliers. Besides, competition, interaction, brainstorming, explanation, questioning, encouragement, and reward systems are presented to enhance the learning motivation to form a climate of teamwork and allow employees of defense suppliers to present a sense of safety and belonging in the activity as well as establish learning motivation and perception of integrity through games, picture books, and experiential activities. Santos Rego et al. (2021) said that teachers should teach online moral education courses in a positive, friendly, and humorous way. In this way, they can develop a trusting interactive mode, and employees will be able to speak freely (Watz, 2011) also mentioned that in order to achieve good learning results, speaking from your mind and explaining the real situation also serves to enhance students’ learning motivation and achievements (Hoedel and Lee, 2016).

## Data Availability Statement

The raw data supporting the conclusions of this article will be made available by the authors, without undue reservation.

## Ethics Statement

The studies involving human participants were reviewed and approved by the Ethical Committee of the I-Shou University, Taiwan. The patients/participants provided their written informed consent to participate in this study.

## Author Contributions

H-CH performed the initial analyses and revised and approved the submitted version of the manuscript.

## Conflict of Interest

The author declares that the research was conducted in the absence of any commercial or financial relationships that could be construed as a potential conflict of interest.

## Publisher’s Note

All claims expressed in this article are solely those of the authors and do not necessarily represent those of their affiliated organizations, or those of the publisher, the editors and the reviewers. Any product that may be evaluated in this article, or claim that may be made by its manufacturer, is not guaranteed or endorsed by the publisher.
